# Fat3 regulates neural progenitor cells by promoting Yap activity during spinal cord development

**DOI:** 10.1038/s41598-022-19029-3

**Published:** 2022-08-30

**Authors:** Soyeon Seo, Young A. Kim, Junekyoung Lee, Seunghwan Lee, Jumee Kim, Seunghee Lee

**Affiliations:** College of Pharmacy and Research Institute of Pharmaceutical Sciences, Seoul National University, Seoul, 08826 South Korea

**Keywords:** Developmental biology, Neuroscience

## Abstract

Early embryonic development of the spinal cord requires tight coordination between proliferation of neural progenitors and their differentiation into distinct neuronal cell types to establish intricate neuronal circuits. The Hippo pathway is one of the well-known regulators to control cell proliferation and govern neural progenitor cell number, in which the downstream effector Yes-associated protein (Yap) promotes cell cycle progression. Here we show that an atypical cadherin Fat3, expressed highly in the neural tube, plays a critical role in maintaining proper number of proliferating progenitors. Knockdown of Fat3 in chick neural tube down-regulates expression of the proliferation markers but rather induces the expression of neural markers in the ventricular zone. We further show that deletion of *Fat3* gene in mouse neural tube depletes neural progenitors, accompanied by neuronal gene expression in the ventral ventricular zone of the spinal cord. Finally, we found that Fat3 regulates the phosphorylation level of Lats1/2, the upstream kinase of Yap, resulting in dephosphorylation and stabilization of Yap, suggesting Yap as a key downstream effector of Fat3. Our study uncovers another layer of regulatory mechanisms in controlling the activity of Hippo signaling pathway to regulate the size of neural progenitor pools in the developing spinal cord.

## Introduction

During the development of vertebrate neural tube, neural stem cells within the ventricular zone proliferate and are patterned to distinct progenitor domains that give rise to different types of neurons and glia, eventually contributing to the assembly of motor circuits^1,2^. The spatio-temporal control of proliferation and differentiation is tightly regulated to maintain tissue homeostasis between progenitors and differentiated cells. Multiple signaling pathways controlling neural progenitor cell number are converged to secure proper tissue size, organization and functioning of the central nervous system (CNS)^3,4^. The Hippo pathway is one of the key regulators controlling cell proliferation and ensuring the number of neural progenitors^5,6^. The vertebrate Hippo pathway kinases such as Mst1/2 and Lats1/2 restrain proliferation by phosphorylating Yap to inhibit its transcriptional activity. Activated Yap interacts with TEA domain transcription factor (TEAD) to promote cell cycle progression, leading to significant expansion of the neural progenitor pools^7^.

The cell surface protocadherin gene *Fat* is known to be a tumor suppresser gene, and Fat acts as an upstream activator of the Hippo pathway^8,9^. Fat family of cadherins are exceptionally large transmembrane proteins that have more than 30 consecutive cadherin repeats as well as laminin A–G domains and EGF repeats in their extracellular domain^10,11^. Fat function is relatively well understood in flies. In *Drosophila*, there are two Fat family cadherins: Fat and Fat-like^10,12^. *Fat* mutations cause overgrowth of larval imaginal discs and planar polarity defects, indicating roles of Fat in Hippo and planar cell polarity (PCP) pathway, respectively^11,13^. It has been shown that another atypical protocadherin, Dachsous (Ds), acts as a ligand that binds to Fat (as a receptor), which forms a dimer and stimulates an intracellular signaling pathway^14,15^. *Four-jointed* (*Fj*), encoding a protein found in transmembrane and as secreted forms, is suspected to act genetically upstream of *Fat-Ds* interactions^16–18^. Although Fat-like proteins have been less studied, they appear to regulate the planar cell polarity in *Drosophila* follicular cells^19^.

Even though Fat3 is present in the nervous system^20,21^, not much has been described for its in vivo function during neurogenesis. The first evidence of Fat3 function in mouse nervous system has been demonstrated in the amacrine cells (ACs) of retina, in which Fat3 was demonstrated to be required for the development of unipolar morphology of ACs. As a polarity protein, Fat3 transmits a local signal that modifies the cytoskeleton organization through its intracellular domain that binds to and localizes Ena/VASP family actin regulators^22,23^. However, the function of Fat3 and its contribution to progenitor cell proliferation and specification remain incompletely understood.

Based on its high expression in the CNS, especially in the spinal cord, we hypothesized a role for Fat3 in progenitor cell proliferation and neuronal specification during embryonic spinal cord development. In the present study, we identify Fat3 as a key regulator in maintaining the appropriate number of proliferating progenitors by enhancing the activity of Yap, a downstream effector of Hippo pathway. Loss of Fat3 function in mouse and chick spinal cord results in down-regulation of proliferation marker genes, while inducing the expression of neural genes in the progenitors. We show that Fat3 interacts with Lats1/2 and inhibits their kinase activity and thus stabilizes the protein level of Yap. Taken together, our data indicate that Fat3 plays crucial roles in the generation and proliferation of progenitor cells and suppression of neural gene expression. Our results also provide critical insights into the multiple cross-talks between Fat proteins and Hippo pathway.

## Results

### Expression of Fat3 in developing mouse and chick spinal cord

**Figure 1:**
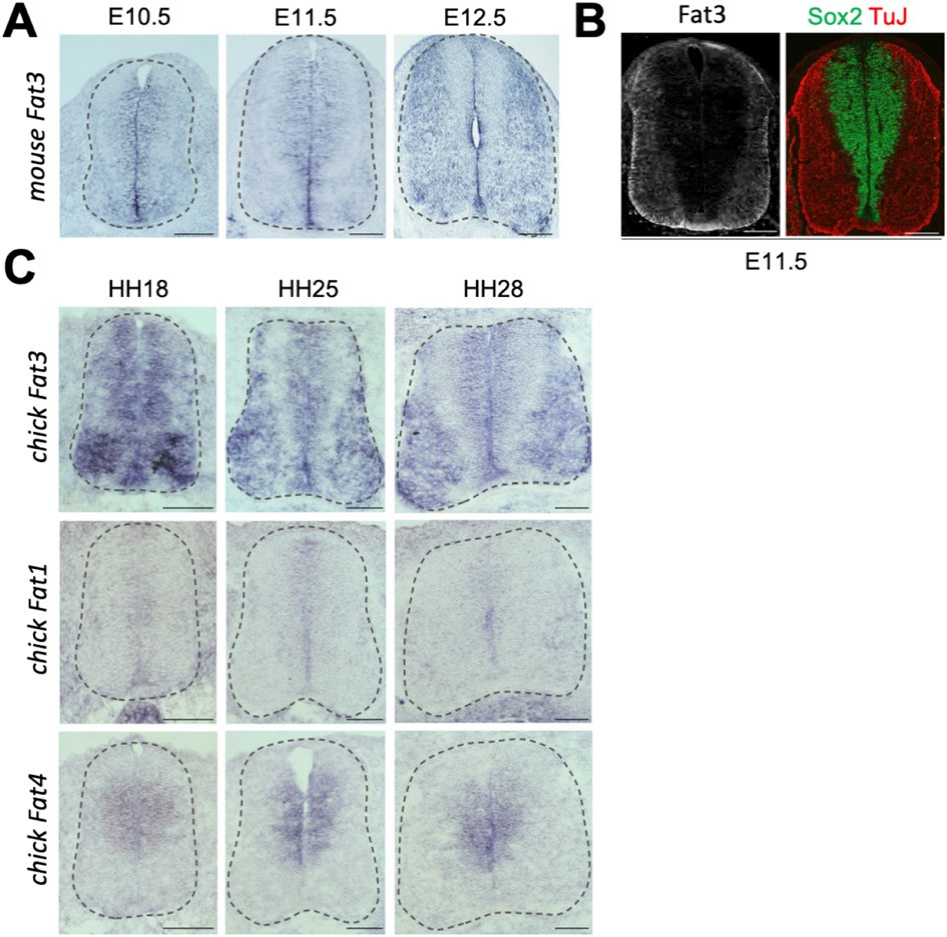
Expression of Fat cadherins in developing spinal cord. (**A**) Expression pattern of Fat3 in mouse embryonic spinal cord by ISH (cervical level). Fat3 is mainly expressed in the ventricular zone and then remained in the mantle zone (MZ) of spinal cord from E10.5–12.5. (**B**) Fat3 protein was detected in the MZ barely at E11.5 by immunohistochemistry (IHC). Sox2 staining shows the neural stem cells and TuJ staining represents differentiated neuronal area. (**C**) Expression patterns of *Fat3, Fat1 and Fat4* in chick spinal cord by ISH*.* Dotted outlines indicate the border of the spinal cord. Scale bars: 100 μm.

To confirm the previous reports and analyze the spatial expression of vertebrate Fat family in the developing spinal cord, we examined the expression of *Fat3* in mouse embryonic spinal cord and also compared the expression pattern of *Fat1*, *Fat3* and *Fat4* in the spinal cord of chick embryos at different developmental stages using in situ hybridization (ISH) (Fig. 1A,C). In the neural tube of developing mouse embryos, the expression of Fat3 was first detected mainly in the apical half of the neural progenitors in the ventricular zone (VZ) at E10.5–11.5 where the progenitor marker Sox2 is expressed (Fig. 1B). Then its expression remained in the neuroepithelial layer and was also detected in the mantle zone (MZ) where neuronal marker TuJ is expressed but absent in the subventricular zone (SVZ) from E12.5 onwards (Fig. 1A). Interestingly, even though the Fat3 antibody was not good enough for mouse tissue staining, we could detect Fat3 protein expression in the MZ barely at E11.5 by immunohistochemistry (IHC) (Fig. 1B). Similar to the expression in mouse spinal cord, chick *Fat3* was also highly expressed in proliferating progenitors in the VZ and then was detected in differentiating neurons in the MZ at Hamburger-Hamilton stages (HH) 18–28 (Fig. 1C). Chick *Fat4* was also detected in the progenitor cells within intermediate regions along the dorsoventral axis of the neural tube as reported^24^ and chick *Fat1* showed relatively weak expression in the midline of the neural tube (Fig. 1C). These expression patterns suggest that Fat3 might function as a major regulator in cell proliferation and neural differentiation within the spinal cord.

### Fat3 regulates progenitor cell proliferation

**Figure 2:**
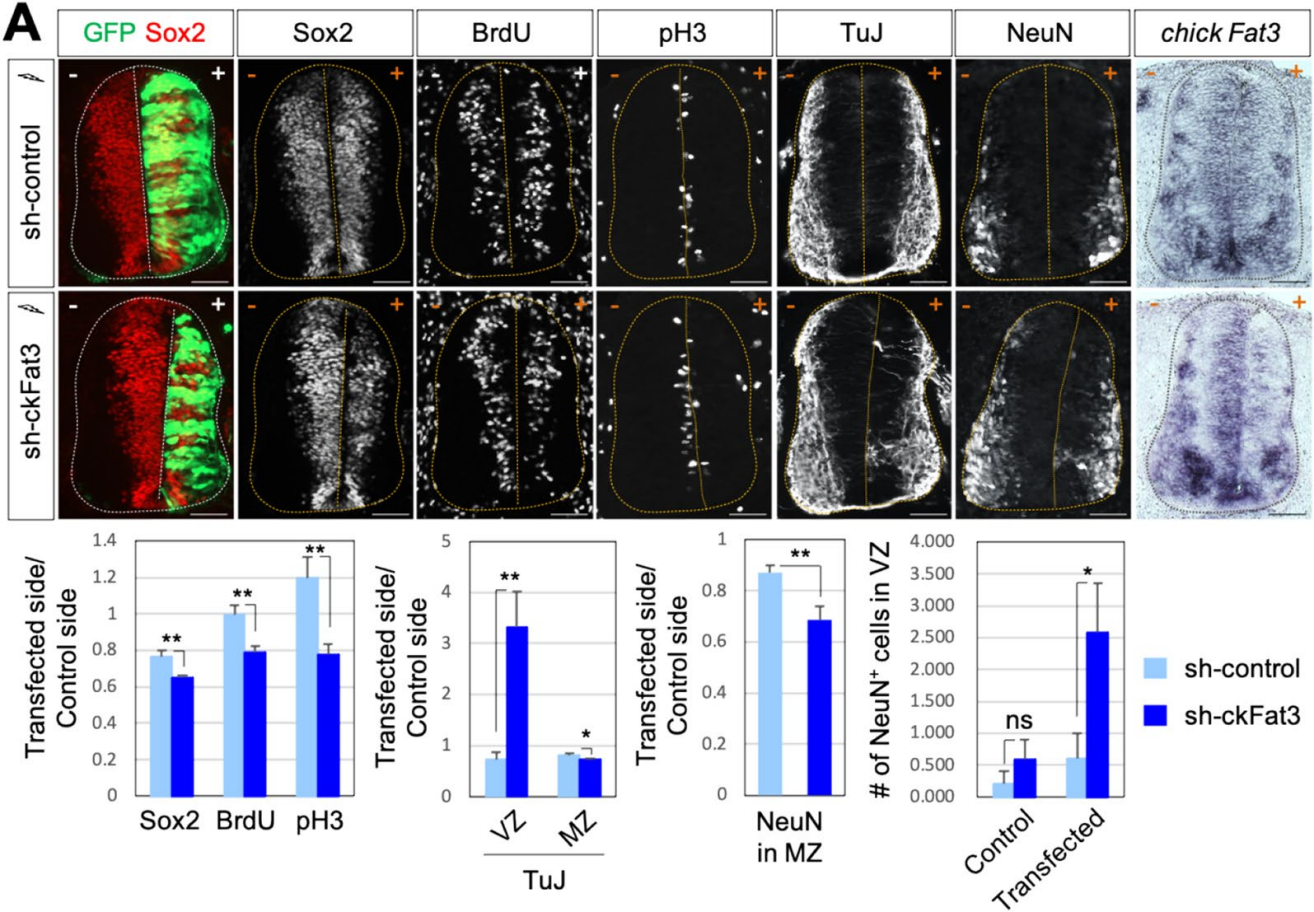
Fat3 is required for proliferation and maintenance of neural stem cell population. (**A**) In ovo electroporation of sh-control or sh-Fat3 was performed at HH13, followed by immunostaining analyses with indicated antibodies for chick embryos (thoracic level). GFP expression indicates transfected cells. Reduction of Fat3 expression by sh-Fat3 knockdown was confirmed by chick *Fat3* ISH. Quantification of Sox2^+^, BrdU^+^, pH3^+^, TuJ^+^ or NeuN^+^ cells in the electroporated side (+) relative to those in the unelectroporated control side (−). Quantification was performed with 15–20 images from 5 to 7 embryos injected with each knockdown construct. Values are means ± sem. *p < 0.05, **p < 0.01 (Student’s *t*-test).; *ns* not significant. Dotted outlines indicate the border of the spinal cord. Scale bars: 100 μm.

To examine the function of Fat3 in spinal cord development, we knocked down *Fat3* in developing chick embryos. We used EFU6-300 knockdown vector containing green fluorescence protein (GFP) to monitor the transfected cells. We first confirmed that Fat3 expression was reduced in the progenitor domains in the VZ and differentiated cells in the MZ in the electroporated side (indicated as + on the right side of the image) compared to the expression in the un-electroporated side (indicated as − on the left side of the image) by sh-ckFat3 knockdown construct but not by sh-control vector using ISH (Fig. 2A). As expected, based on the expression pattern of Fat3 in the proliferating cells, the number of Sox2, BrdU and phosphohistone 3 (pH3) positive proliferating cells was significantly reduced by knockdown of *Fat3* gene in developing chick neural tube compared to the un-electroporated control side (Fig. 2A), which resulted in a mild shrunken neural tube because of the loss of progenitors on the transfected side. Interestingly, the expression of pan-neuronal gene β-tubulin III, as labeled by TuJ-staining, was significantly induced in the VZ of the spinal cord by *Fat3* knockdown. Consistent with the ectopic TuJ expression, the number of the neuronal marker NeuN^+^ cells in the VZ of the *Fat3* knockdown embryo was increased compared to the control knockdown embryo in the transfected side (Fig. 2A), indicating that loss of Fat3 facilitates expression of neural markers in the prospective neurons, while their nuclei have not yet settled in the marginal zone. Moreover, the number of neurons in the MZ was also reduced by sh-Fat3 knockdown compared to the control side, that might be caused by the depletion of neural progenitors and/or by the impaired migration of new-born neurons located in the VZ. These results suggest that Fat3 plays crucial roles in promoting cell proliferation and regulating neuronal differentiation in the developing spinal cord.

### Deletion of *Fat3* in the developing mouse spinal cord causes defects in progenitor cell proliferation

**Figure 3:**
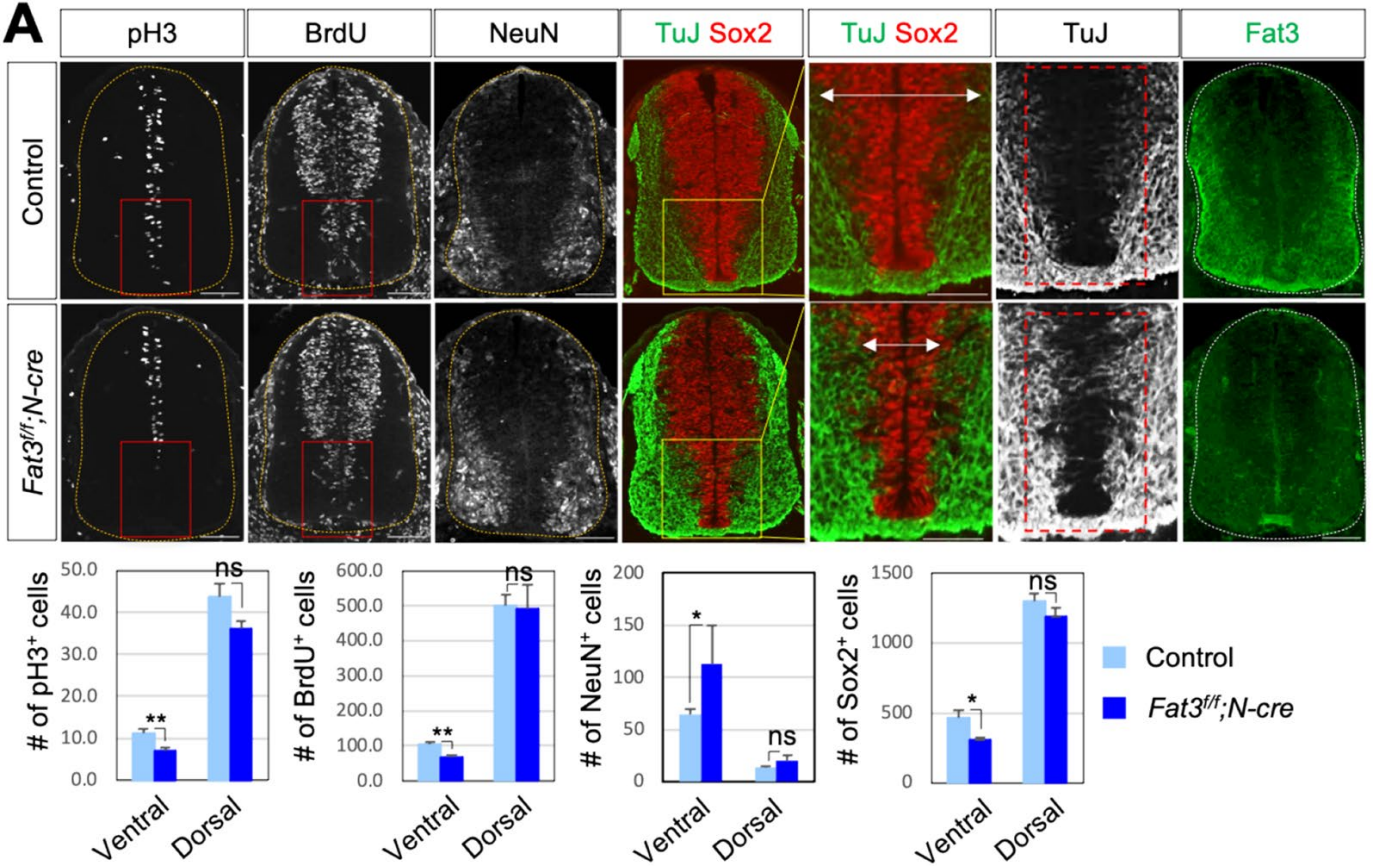
Fat3 is crucial for proper ventral progenitor formation in developing mouse spinal cord. (**A**) The expression of proliferation markers (pH3, BrdU, Sox2) and neuronal markers (NeuN and TuJ) in transverse sections at E11.5 in *Fat3*^*f/f*^*;N-Cre* embryos (n = 3) and their littermate controls (n = 4) by immunostaining analyses with indicated antibodies for mouse embryos (cervical level). IHC with anti-Fat3 antibody confirms the absence of Fat3 expression in *Fat3*^*f/f*^*;N-Cre* embryos. Quantification was performed with 5–10 images from multiple embryos as indicated and at least three sections from each embryo. Values are means ± sem. *p < 0.05, **p < 0.01 (Student’s t-test); *ns* not significant. Dotted outlines indicate the border of the spinal cord. Scale bars: 100 μm.

Our findings suggest that loss of Fat3 in proliferating progenitors impairs their proliferation. To further validate these results, we analyzed the embryonic spinal cord of mice deficient in *Fat3*. To delete Fat3 specifically in the developing spinal cord, we bred mice carrying floxed *Fat3* allele with *Nestin*-Cre mice (designated as *N*-Cre), which express Cre-recombinase in neuroblasts^25^. By IHC with an anti-Fat3 antibody, we confirmed that Fat3 protein is not expressed in MZ of the *Fat3*^*f/f*^;*N*-cre mutant mouse spinal cord at embryonic stage 11.5 (E11.5) (Fig. 3A). First, to analyze whether Fat3 is required for the proliferation of the neural progenitors, similar to our chick results (Fig. 2), we performed IHC with pH3 and BrdU labeling at E11.5 (Fig. 3A). Interestingly, compared with littermate control embryos, *Fat3*^*f/f*^;*N*-cre embryos showed a decrease of pH3^+^ cells and BrdU labeling and a reduction of Sox2^+^ progenitor cells in the ventral ventricular zone, whereas TuJ expression was induced in the ventral ventricular area, where ventral interneurons and motor neurons (MNs) are generated (Fig. 3A), suggesting that Fat3 is likely to be involved in cell proliferation, cell cycle exit and neuronal differentiation in ventral progenitor cells. There was no significant effect in dorsal area and this might be due to the redundant function of other Fat family expressed in the intermediate progenitors. We detected no significant cell death within the spinal cord of *Fat3* mutants as shown by IHC with cleaved caspase 3 antibody (data not shown).

### Fat3 controls the Yap protein stability and activity

Fat family members such as *Drosophila* Fat and Fat4/FatJ control tissue size and cell proliferation through the Hippo signaling pathway^24^. Hippo pathway controls the number of neuronal progenitors in the neural tube by modulating cell proliferation and apoptosis^5^. Yap, a Hippo downstream effector, acts as a transcriptional coregulator of Tead transcription factor that induces Cyclin D1 for cell proliferation. The stability and activity of Yap is negatively regulated by the upstream Hippo pathway kinases including Mst1/2 and Lats1/2^9,26^. In addition, Fat4 acts upstream of Yap and negatively regulates Yap activity, as Fat4 knockdown decreases the level of phosphorylated Yap, increasing active form of Yap, and thus the increased number of interneurons by Fat4 knockdown in the chick neural tube is rescued by knockdown of Yap^24^. Given these results, we decided to test whether the molecular mechanism of Fat3 in controlling the cell proliferation and neurogenesis is involved in the Hippo signaling pathway.

**Figure 4:**
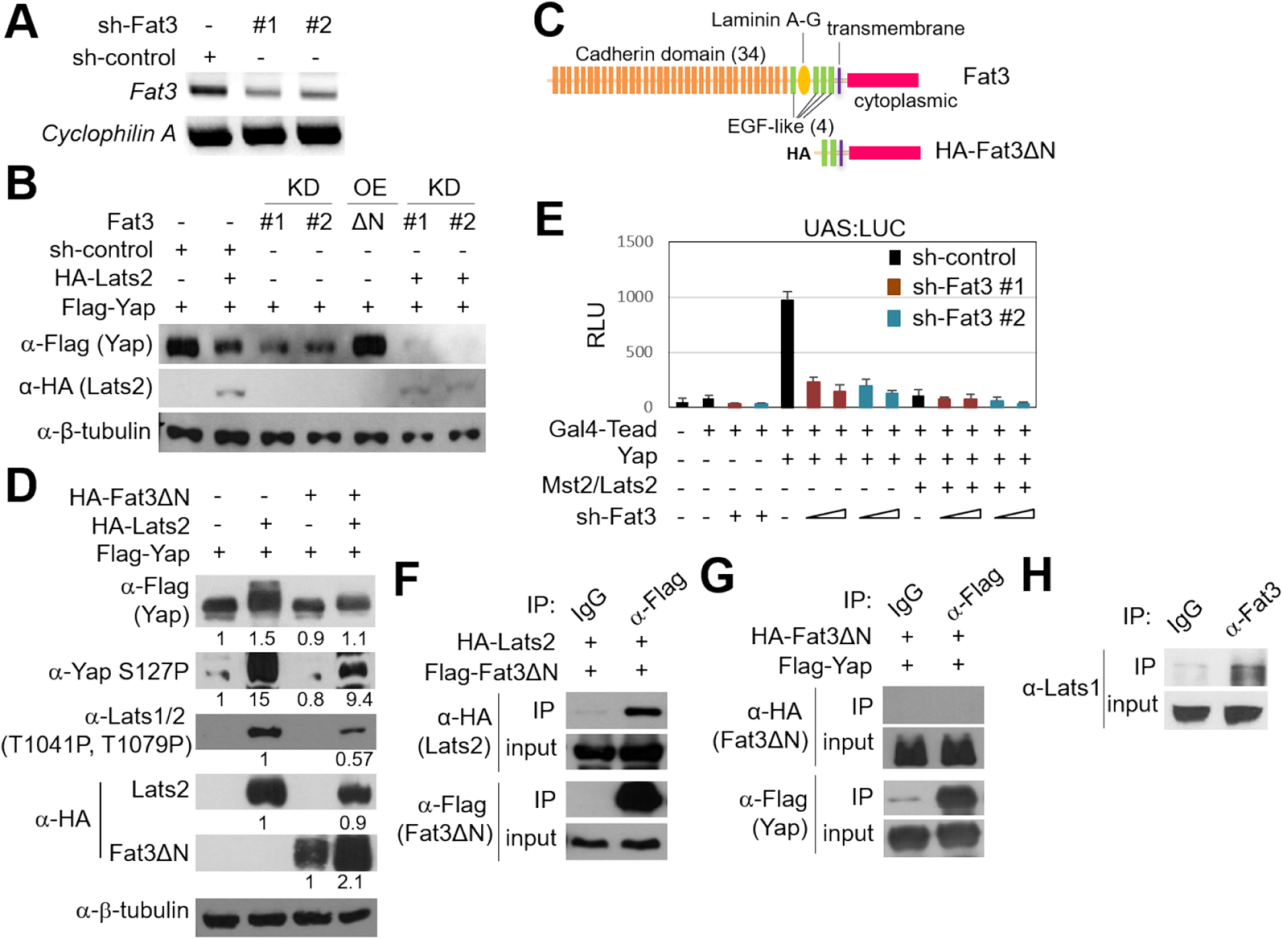
Fat3 knockdown destabilizes and attenuates Yap transcriptional activity. (**A**) P19 cells were transfected with sh-control and sh-Fat3 (target sites #1 and #2), followed by RT-PCR to test knockdown efficiency. (**B**) P19 cells were transiently transfected with sh-Fat3 and Fat3ΔN together with Flag-Yap, HA-Lats2 and HA-Mst2. After 48 h, cell lysates were analyzed by western blotting. (**C**) Schematic representation of the mouse Fat3 full length and N-terminus deleted construct of Fat3 (ΔN). (**D**) The phosphorylation level of Yap by Lats2/Mst2 was attenuated by overexpression of Fat3ΔN. anti-S127P Yap antibody detects the phosphorylated Serine 127 residue of Yap. anti-T1041P, T1079P Lats1/2 antibody detects the phosphorylated Lats2 on Thr1041 and Thr1079. Quantification of each signal measured by ImageJ and then normalized by tubulin is shown below each band. (**E**) Luciferase reporter assay with UAS:LUC and Gal4-Tead showed the transcriptional activity augmented by Yap, that was abrogated by Fat3 knockdown. Values are means ± sd. At least 3 replicate experiments were repeated in P19 cells. (**F,G**) Fat3 interacts with Lats2 but not with Yap. Co-immunoprecipitation assay with HEK293T cells transiently transfected with the expression vectors for HA-tagged Lats2 and Flag-tagged Fat3ΔN (**F**), and HA-tagged Fat3ΔN and Flag-tagged Yap (**G**), respectively. (**H**) CoIP assay with E11.5 mouse spinal cord extract showed interaction between Fat3 and Lats1. To detect same samples with different antibodies, membranes were cut prior to hybridization or reprobed. Uncropped original western blot images are included in the “Supplementary Data”, with cropped areas highlighted with colored boxes.

To test if Fat3 affects the protein stability of Yap, we checked the protein level of Yap in the condition of Fat3 knockdown in P19 mouse embryonic carcinoma cells that were harvested 48 h after transfection. First, the reduced expression of Fat3 by knockdown constructs (target sites, #1 and #2) was confirmed by RT-PCR (Fig. 4A). As previously reported, the level of Yap was decreased by Lats2 and Mst2 kinases that phosphorylate and induce proteasomal degradation of Yap (Fig. 4B, lane 2). Interestingly, the protein level of Yap was dramatically decreased by Fat3 knockdown constructs (Fig. 4B, lane 3 and 4), which further facilitates the reduction of Yap protein level in the presence of Lats2 (Fig. 4B, lane 6 and 7), suggesting that Fat3 prevents the degradation of YAP protein. As the full length *Fat3* gene encodes a single-pass transmembrane protein with huge extracellular cadherin domains and intracellular domains of 4555 aa, it is not easy to overexpress the full length Fat3 in cell lines. Given that the effect of Fat family proteins is mediated through the intracellular domain, we employed the N-terminus deleted Fat3 (Fat3ΔN) (Fig. 4C). The overexpression of Fat3ΔN did not facilitate or prevent the degradation of Yap protein (Fig. 4B, lane 5). To further examine the regulation of the Hippo pathway by Fat3, we next compared the levels of phosphorylated Yap (pYap) after Fat3ΔN overexpression. Human embryonic kidney 293T (HEK293T) cells were harvested 24 h after transfection to monitor the phosphorylation of Yap before it is degraded, and western blot analysis showed the shifted band of Yap, indicative of phosphorylation of Yap, in the presence of Lats2/Mst2, and this shifted Yap band disappeared by Fat3ΔN (Fig. 4D, lane 2 and 4). Moreover, immunoblotting with an antibody specifically detecting phosphorylated serine 127 of Yap (Yap S127P) confirmed a decrease in serine 127-phosphorylated Yap in the presence of Fat3ΔN (Fig. 4D, lane 4). Of note, we also found that the levels of Lats2 protein and Lats1/2 phosphorylation (Lats1/2 T1041P, T1079P) were reduced by Fat3ΔN (Fig. 4D), indicating that Fat3 inhibits the Lats activity.

Next, we tested whether Fat3 affects the transcriptional activity of Yap using Gal4-UAS luciferase system. Expression of Yap stimulated the luciferase reporter activity driven by Gal4-Tead, while Gal4-Tead alone showed little activity. Moreover, the reporter activity was significantly dampened by shRNA against Fat3 (Fig. 4E), indicating that Fat3 enhances the activity of Yap possibly through dephosphorylating and stabilizing the protein level of Yap.

To elucidate the molecular mechanism of how Fat3 facilitates Yap activity, we tested the possibility of Fat3 to interfere with the Hippo pathway or to activate Yap directly. First, we performed coimmunoprecipitation (coIP) assays to test whether Fat3ΔN and Lats2 interact with each other using HEK293T cells expressing HA-tagged Lats2 and Flag-tagged Fat3ΔN. HA-Lats2 was coimmunoprecipitated with Flag-Fat3ΔN by anti-Flag antibody (Fig. 4F). However, HA-tagged Fat3ΔN was not coimmunoprecipitated with Flag-Yap (Fig. 4G). To further test whether Fat3 interacts with Lats kinases in vivo, we performed coIP using spinal cord cell lysate from E11.5 mouse embryos. Indeed, we could detect Lats1 interacts with Fat3 in mouse neural tube (Fig. 4H). These results indicate that Fat3 affects Lats1/2 phosphorylation through interacting with Lats1/2, which indirectly results in positive regulation of the Yap activity.

### Yap is sufficient to rescue the reduction of neural progenitors caused by loss of Fat3

Given the expression pattern of Fat3 and Yap in the developing spinal cord (Fig. 1)^5^ and our findings for stabilization and activation of Yap by Fat3 (Fig. 4), we considered the possibility that Yap mediates the effect of Fat3 in controlling progenitor cell proliferation.

**Figure 5:**
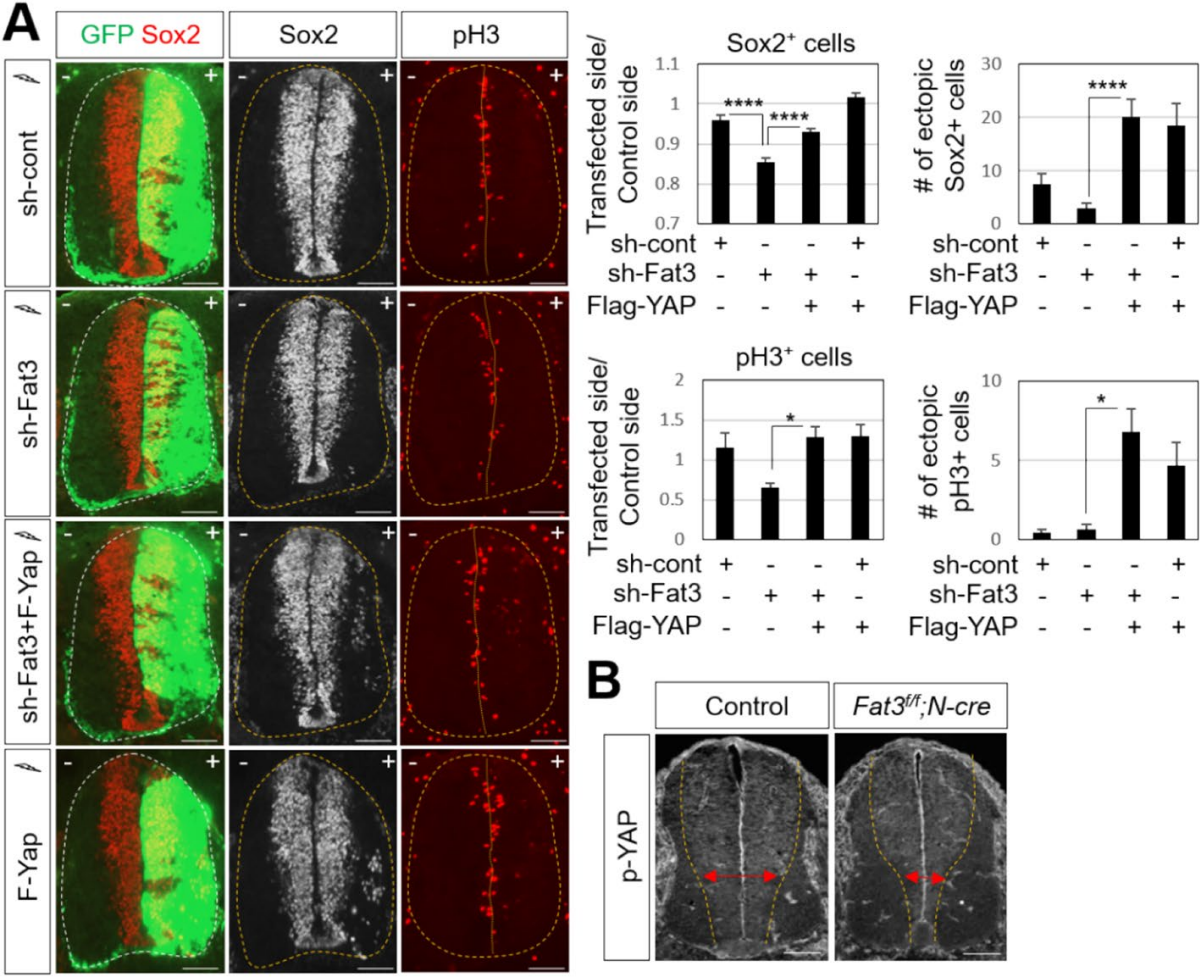
Overexpression of Yap recovers Fat3 loss-of-function effect. (**A**) In ovo electroporation of Flag-Yap together with sh-Fat3 was performed at HH13, followed by immunostaining analyses with indicated antibodies for chick embryos (thoracic level). +, electroporated side. GFP expression indicates transfected cells. Quantifications of Sox2^+^ and pH3^+^ cells in the electroporated side (+) relative to those in the unelectroporated control side (−) and the number of ectopic Sox2^+^ and pH3^+^ cells in the electroporated side were shown. Quantification was performed with 9–15 images from 4 to 6 embryos of each test set. Values are means ± sem. Statistical difference was determined by one-way ANOVA followed by Turkey’s test using Prism 9 (GraphPad) software; *p < 0.05, ****p < 0.0001. Scale bars: 100 μm. (**B**) Immunostaining analyses with anti-phospho Yap antibody showed reduced progenitor domain in the ventral ventricular zone in E11.5 *Fat3*^*f/f*^*;N-Cre* embryos compared to their littermate controls. Scale bars: 100 μm.

To test whether Yap acts downstream of Fat3, Yap construct was co-electroporated with the *Fat3* knockdown construct. We found that the reduction of Sox2^+^ and pH3^+^ cell numbers by *Fat3* knockdown in chick neural tubes was rescued by overexpressing Yap (Fig. 5A). Furthermore, Yap overexpression resulted in ectopic induction of Sox2 (around 20 Sox2^+^ cells), and pH3 (around 7 pH3^+^ cells) in the lateral side of the neural tube even in the condition of *Fat3* knockdown. Next, to further confirm whether the defect of neural progenitor proliferation in *Fat3*^*f/f*^;*N*-cre embryos was caused by the loss of Yap, we monitored the level of phospho Yap (p-Yap). The domain of p-Yap matched the progenitor zone, indicating that the Hippo signaling pathway is active in neural progenitor cells within the neural tube. Although there was no difference in the signal intensity of p-Yap within cells, the domain expressing p-Yap in the ventral ventricular area was significantly reduced in *Fat3*^*f/f*^;*N*-cre embryos (Fig. 5B), consistent with the reduction of Sox2^+^ progenitor cells (Fig. 3). These results suggest that Fat3 acts through Hippo signaling downstream effector, Yap, to control the proliferation and differentiation within the developing spinal cord.

### The function of Fat3 in regulating neural progenitor proliferation is mediated by Lats kinases

**Figure 6:**
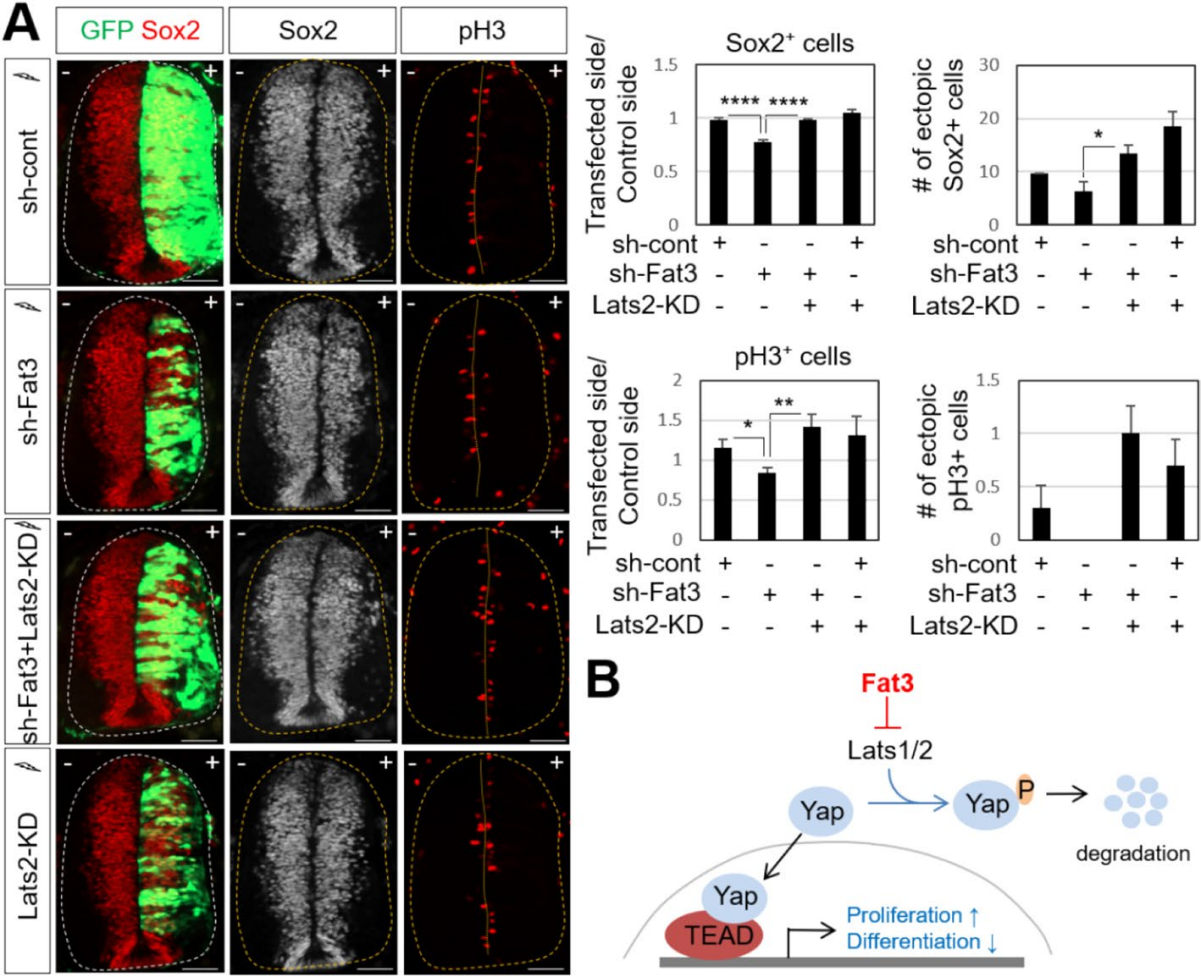
Overexpression of Lats2-KD rescues the proliferation defect caused by Fat3 knockdown. (**A**) In ovo electroporation of Myc-Lats2-KD together with sh-Fat3 was performed at HH13, followed by immunostaining analyses with indicated antibodies for chick embryos (thoracic level). +, electroporated side. GFP expression indicates transfected cells. Quantifications of Sox2^+^ and pH3^+^ cells in the electroporated side (+) relative to those in the unelectroporated control side (−) and the number of ectopic Sox2^+^ and pH3^+^ cells in the electroporated side were shown. Quantification was performed with 9–10 images from 4 to 6 embryos of each test set. Values are means ± sem. Statistical difference was determined by one-way ANOVA followed by Turkey’s test using Prism 9 (GraphPad) software; *p < 0.05, ****p < 0.0001. Scale bars: 100 μm. (**B**) Working model. Fat3 promotes cell proliferation and limits neurogenesis through enhancing Yap transcriptional activity. The phosphorylation of Yap by Lats1/2 and the degradation of Yap was inhibited by Fat3. The increased Yap protein localizes in the nucleus and enhances the activity of Tead transcription factor and induces the expression of proliferation genes.

It has been reported that Mst1/2 and Lats1/2, the upstream kinases of the Hippo pathway, regulate the proliferation and survival of neural progenitors by suppressing Yap activity^5^. Given that Yap overexpression recovers the decrease of neural progenitors by Fat3 knockdown and Lats kinases act as key modulators of Yap activity, we investigated whether inhibition of Lats kinases can also rescue neural progenitor proliferation defect caused by Fat3 loss of function. To inhibit Lats2 function, we used a kinase-dead version of Lats2 (Lats2-KD) that had been shown a dominant-negative effect^5,27^. Lats2-KD construct was co-electroporated with the *Fat3* knockdown construct. We observed that the reduction of Sox2^+^ and pH3^+^ cell numbers by *Fat3* knockdown compared to the control side was recovered by overexpressing Lats2-KD (Fig. 6A), although the ectopic induction of Sox2 and pH3 in the lateral side of the neural tube was not significant when Lats2-KD alone was expressed. Lats2-KD showed less effect in over-proliferation as compared to that resulted by overexpressing Yap. We assumed that this approach may have only partially decreased the activities of endogenous Lats kinases, indicating that there may be less active Yap than direct overexpression of Yap. These results support our model that Fat3 inhibits Lats kinases to stimulate Yap activity, ultimately promoting progenitor cell proliferation in the neural tube.

## Discussion

In this report, we presented our findings that the atypical cadherin Fat3 plays crucial roles in proliferation and differentiation of neural progenitor cells in the spinal cord. We showed that Fat3 is expressed in the proliferating progenitor cells at early developmental stages and also detected in the differentiated neuronal cells including motor neurons in the mantle area at later developmental stages of mouse and chick spinal cords. Fat3 functions to enhance proliferation and inhibit differentiation of neural progenitors, which interestingly is the opposite of the functions known for its other family members Fat1 and Fat4^24,28^. We propose a model in which Fat3 interacts with and suppresses the protein levels and the kinase activity of Lats1/2, thereby stabilizing the downstream effector Yap (Fig. 6B). We further propose that this is important for maintaining the appropriate size of neural progenitor cell pool, and that fine-tuning the balance between different Fat cadherin activities is critical for maintaining proper neural progenitor proliferation and neurogenesis.

Mammals have four Fat family of cadherins, Fat1, Fat2, Fat3, and Fat4/Fat-J. Fat1–3 are relatively similar to each other, and resemble *Drosophila* Fat-like, while Fat4/Fat-J is considered to be the ortholog of *Drosophila* Fat (dFat). In *Drosophila*, dFat activates Hippo pathway and prevents the expression of Cyclin E and Diap1 that promotes cell proliferation and inhibits apoptosis^29^, thereby suppressing excessive tissue growth. This Hippo pathway is well conserved in vertebrates and involved in growth control. Fat1, 3 and 4 are expressed in the brain and spinal cord during embryonic development, whereas Fat2 is expressed only in adult granule cells of the cerebellum^18,20,21^. It has been shown that Fat1 and Fat4/Fat-J play crucial roles in mouse brain development. Loss of Fat1 and Fat4/Fat-J in mouse embryos results in neural tube closure defects, increased neural progenitor proliferation, and altered apical constrictions, possibly through Fat1 and Fat4 heterodimer formation^28^. Moreover, loss of Fat1 and Fat4 in murine cortices displays enhanced radial precursor proliferation and delayed cell cycle exit^28^. In addition, holoprosencephaly and anophthalmia are observed in *Fat1* deficient mice^30^. Although these midline defects may result from defective cell–cell communications during early development, the detailed action of Fat1 in these pathways remains unclear. Fat4 regulates the Hippo pathway mediator Yap1 negatively and restricts the size of the progenitor pools, thereby contributing to providing appropriate numbers of distinct interneuron subtypes in the neural tube^24^. Fat4/Fat-J restricts the size of specific subsets of neural progenitor pool in chick spinal cord by acting through the Hippo pathway. Loss of Fat-J in chick spinal cord results in an increase in dp4-vp1 progenitors, which is rescued by simultaneous deletion of the Yap^24^. Mouse *Fat4*^*−/−*^ embryos show a wider spinal cord than control littermates^31^. Given these results, our findings for the functions of Fat3 in enhancing proliferation and inhibiting differentiation of neural progenitors were rather surprising. In this regard, it is notable that mutations of many of the components in the Hippo pathway have been identified in human cancers including Yap in medulloblastoma. *FAT* genes are relatively frequently mutated in several human cancers suggesting actions as either tumor suppressors or tumor promoters, based on other physiological functions of FATs. It will be interesting to further test whether Fat3 can associate with and modulate Fat1 or Fat4 activity and ultimately regulates Hippo pathway indirectly.

Our analysis of the loss-of-function phenotypes for Fat3 provide crucial insights into novel actions of cadherin proteins in early CNS development and suggest mechanisms regulating the proportion of proliferating progenitor pools vs. differentiated neurons. Interestingly, loss-of-function for Fat3 leads to not only decreasing the progenitor cell number but also facilitating the expression of neural markers in the VZ. These results suggest that Fat3 functions to maintain the appropriate progenitor pool size and directly inhibits the precocious neural gene expression in neural progenitors. Deletion of *Fat3* in mouse embryonic neural progenitors confirmed the activity of Fat3 to regulate the proliferation and maintenance of neural progenitors, although the effect was detected only in the ventral VZ not in the dorsal side of spinal cord (Fig. 3). We can speculate that other cadherins or Fat-like proteins expressed in the dorsal progenitors might compensate for the loss of Fat3. Reduction of neural progenitors led to the simultaneous increase of TuJ expression in ventral VZ, which was consistent with the results caused by *Fat3* knockdown in chick spinal cord. Although *Fat3* deletion promoted neural gene expression in the ventral VZ, the overall number of ventral motor neurons (MNs) and interneurons (INs) in *Fat3*-cKO was not changed compared to that in control littermates (data not shown). Further studies need to determine the detailed molecular basis of the specific role of Fat3 in ventral progenitor domains and define the potential action of Fat3 in differentiated neurons at later developmental stages.

Intriguingly, our data demonstrate that Fat3 maintains the proper size of progenitor cell pool by inhibiting the Hippo signaling pathway. Our results revealed that Fat3 suppresses the levels of Lats2 proteins and the phosphorylation of Lats1/2 at T1041 and T1079 leading to stabilization of Yap. The T1041/T1079 residues of Lats1/2 are phosphorylated by Mst1/2 and mitogen-activated protein kinase kinase kinase kinase (MAP4K) family kinases, leading to the activation of Lats2 kinase activity^32,33^. It remains to be determined whether the reduced level of T1041/T1079 phosphorylation by Fat3 is simply due to the lower level of Lats1/2 proteins or Fat3 directly inhibits phosphorylation of Lats1/2 at T1041/T1079 by Mst1/2 and MAP4K. Since Mst1/2 interact with and stabilize their kinase substrates^9,34^, it is possible that Fat3 interactions with Lats1/2 may interfere with its phosphorylation by Mst1/2 and MAP4K. Future studies will be directed at elucidating the molecular basis for how Fat3 modulates the proteins levels and phosphorylation of Lats1/2. Lats1/2 have been shown to function as the key regulatory factor of the Hippo pathway in mammals as they play essential roles in directing Yap regulation by various signaling cues affecting the transcriptional activity of Yap. Based on the effect of Fat3 in reducing Lats1/2 phosphorylation and activation, we propose that Fat3 participates as a crucial component of the Hippo pathway at least in part by impinging on Lats1/2.

In summary, we showed that atypical cadherin Fat3 plays critical roles in proliferation and maintenance of progenitor cells by inhibiting the Hippo pathway component Lats1/2, thereby stimulating its downstream effector Yap. Activated Yap together with TEAD transcription factor induces the expression of genes involved in cell proliferation and also inhibits the expression of genes involved in differentiation (Fig. 6B). Together, our data suggest that Fat3 mediates novel regulatory mechanisms to acquire proper numbers of cells in progenitor pool and ensure the timely differentiation of neural progenitors to neurons in the neural tube.

## Materials and methods

### DNA constructs

Mouse *Fat3* deletion construct (ΔN) was cloned into pCS2 containing HA or Flag-epitope tag, Flag-Yap and Myc-Lats2-KD in pCMV, HA-Lats2 and HA-Mst2 in pcDNA3 vector for expression in mammalian cells and chick embryos, as previously described^7^. For knockdown of Fat3 and Yap in chick embryos and Fat3 in mouse P19 cells, shRNA constructs against chick and mouse Fat3 were prepared in EFU6-300 vector, which contains GFP to monitor transfected cells. shRNA construct against chick Yap was prepared in hU6-pBS vector. The shRNA-targeting sequences were as follows: 5′-ATA CAG AGC AAG TGT CAA A-3′ for sh-chick Fat3, 5′-AAC AGG GAA ATC CAA GAC A-3′ for sh-mouse Fat3 #1, 5′-GTG AAT CAG AGA TCA CAG C-3′ for sh-mouse Fat3 #2 and 5′-CTG AGG ACT ATG ACT ACA AAT A-3′ for sh-chick Yap.

### In ovo electroporation, IHC and ISH assays

In ovo electroporation and immunohistochemistry were performed as described^35^. DNAs were injected into the lumen of chick neural tube at HH stage 12 and then electroporated. The embryos were harvested after 2 or 3 days later, fixed in 4% paraformaldehyde, embedded in OCT and cryosectioned in 12 μm thickness for IHC assays or 18 μm thickness for ISH. Each set of chick electroporation experiment was repeated at least three times with more than 5 embryos injected with each combination of plasmids. Similar results along the rostral-caudal spinal cord of the chick embryo were produced in IHC and ISH analyses.

For IHC assays, the following antibodies were used; rabbit anti-Sox2 (Abcam), mouse anti-TuJ (Covance), mouse anti-HA (Covance), goat anti-LacZ (Abcam), mouse anti-phospho H3 (Cell Signaling), mouse anti-BrdU (Sigma-Aldrich), mouse anti-NeuN (Millipore), rabbit anti-phospho YAP (Cell Signaling) and rabbit anti-Fat3^22^.

For ISH analyses, cDNAs for mouse *Fat3*, *chick Fat3, Fat1, Fat4, Yap* were cloned to pBluescript vector to generate digoxigenin-labeled riboprobes.

### Mice

The mice were maintained according to the guidelines of Seoul National University Animal Experiment Ethics Committee. All animal experiments were in accordance with animal welfare laws, complied with ARRIVE guidelines and protocols approved by the Institutional Animal Care and Use Committee of the Institute of Laboratory Animal Resources, Seoul National University (Institutional Animal Care and Use Committee permit number: SNU-161115-5). All methods were carried out in accordance with relevant guidelines and regulations. The generation of *Fat3*^*f/f*^ and *Nestin-Cre* mice has been described previously^22,25^. *Fat3*^*f/f*^ mice were crossed with *Nestin-Cre lines (designated as N-Cre)* for analyses. Mouse embryos were collected at the indicated developmental stages, and processed similarly to chick embryos as described above. Cervical, brachial and thoracic levels of the mouse embryo were used for IHC.

### Image analysis and quantification

Zeiss Axio imager was used to image in situ hybridization and immunohistochemistry results. For quantification of IHC images, serial sectioning was performed on embryos. Stage matched sections with same anterior–posterior level were used to compare controls and mutants. Statistical differences were determined by Student’s *t*-test and one-way analysis of variance (ANOVA) followed by Tukey’s test using Prism 9 (GraphPad Software, La Jolla, CA) software. Statistical significance is displayed as *p* < 0.05 (one asterisk), *p* < 0.01 (two asterisks) or *p* < 0.0001 (four asterisks). ‘ns’ indicates not-significant (*p* > 0.05).

### Co-immunoprecipitation assays and immunobloting assays

HEK293T cells from our lab (gift from JW. Lee at UB) were cultured in DMEM media supplemented with 10% fetal bovine serum (FBS). For coimmunoprecipitation, HEK293T cells were seeded on 10 cm tissue culture dishes, cultured in DMEM media supplemented with 10% FBS and transfected with the expression vectors for HA-Lats2, Flag-Fat3ΔN, HA-Fat3ΔN and Flag-Yap, using Superfect (Qiagen) and harvested 24 h after transfection. P19 cells were seeded in 6-well plates and indicated plasmids were transfected using Lipofectamine 2000 (Invitrogen) and harvested 48 h after transfection. Embryonic day 11.5 mouse embryos were harvested and spinal cord was isolated from the embryo. 20 spinal cords were pooled and used for each immunoprecipitation. Harvested cells were lysed in IP buffer (20 mM Tris–HCl, pH 8.0, 0.5% NP-40, 1 mM EDTA, 150 mM NaCl, 2 mM PMSF, 10% Glycerol, 4 mM Na3VO4, 200 mM NaF, 20 mM Na-pyroPO4, and protease inhibitor cocktail). In these studies, immunoprecipitations were performed with mouse anti-Flag (Sigma-Aldrich) and rabbit anti-Fat3 antibodies. Western blotting assays were monitored using rabbit anti-Yap S127P (Cell Signaling), rabbit anti-phospho-Lats1/2 (T1041, T1079) (Invitrogen), rabbit anti-Lats1 (Cell Signaling), mouse anti-HA (Covance), mouse anti-Flag (Sigma-Aldrich) and rabbit anti- β-tubulin (SantaCruz). Uncropped original western blot images are included in the “Supplementary Data”, with cropped areas highlighted with colored boxes.

### Luciferase assays

For the luciferase reporter assay, P19 cells were seeded in 48-well plates. UAS-luciferase reporter, CMV-β-gal, and indicated plasmids were co-transfected using Lipofectamine 2000 (Invitrogen). Forty-eight hours after transfection, cells were lysed and cell extracts were assayed for luciferase activity and the luciferase values were normalized to β-galactosidase activity.

## Supplementary Information

Supplementary Figures.
