# Supplementary material for: Fat3 regulates neural progenitor cells by promoting Yap activity during spinal cord development
Source: Sci Rep. 2022 Aug 30;12:14726. doi: 10.1038/s41598-022-19029-3 (PMC9427758; doi:10.1038/s41598-022-19029-3)
Supplement: Supplementary file 1 — Supplementary Figures. [file 41598_2022_19029_MOESM1_ESM.docx]

**Supplementary information**

**Fat3 governs neural progenitor number by dephosphorylating and stabilizing Yap during spinal cord development**

Soyeon Seo^1^, Young A. Kim^1^, Junekyoung Lee^1^, Seunghwan Lee^1^, Jumee Kim^1^ and Seunghee Lee^1,*^

^1^College of Pharmacy and Research Institute of Pharmaceutical Sciences, Seoul National University, Seoul 08826, Korea


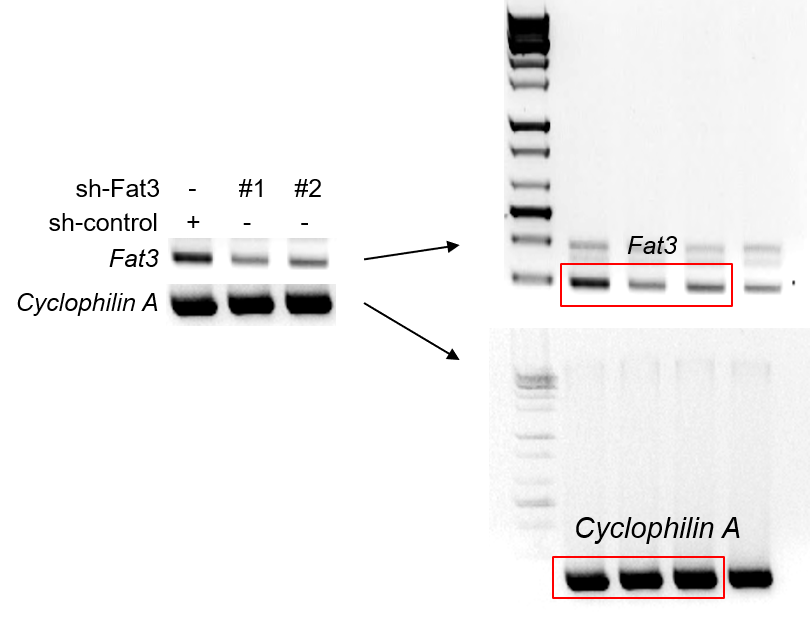


Figure S1: Full length Gels of *Fat3* and *Cyclophilin A* in RT-PCR (shown as cropped images in Figure 4A).


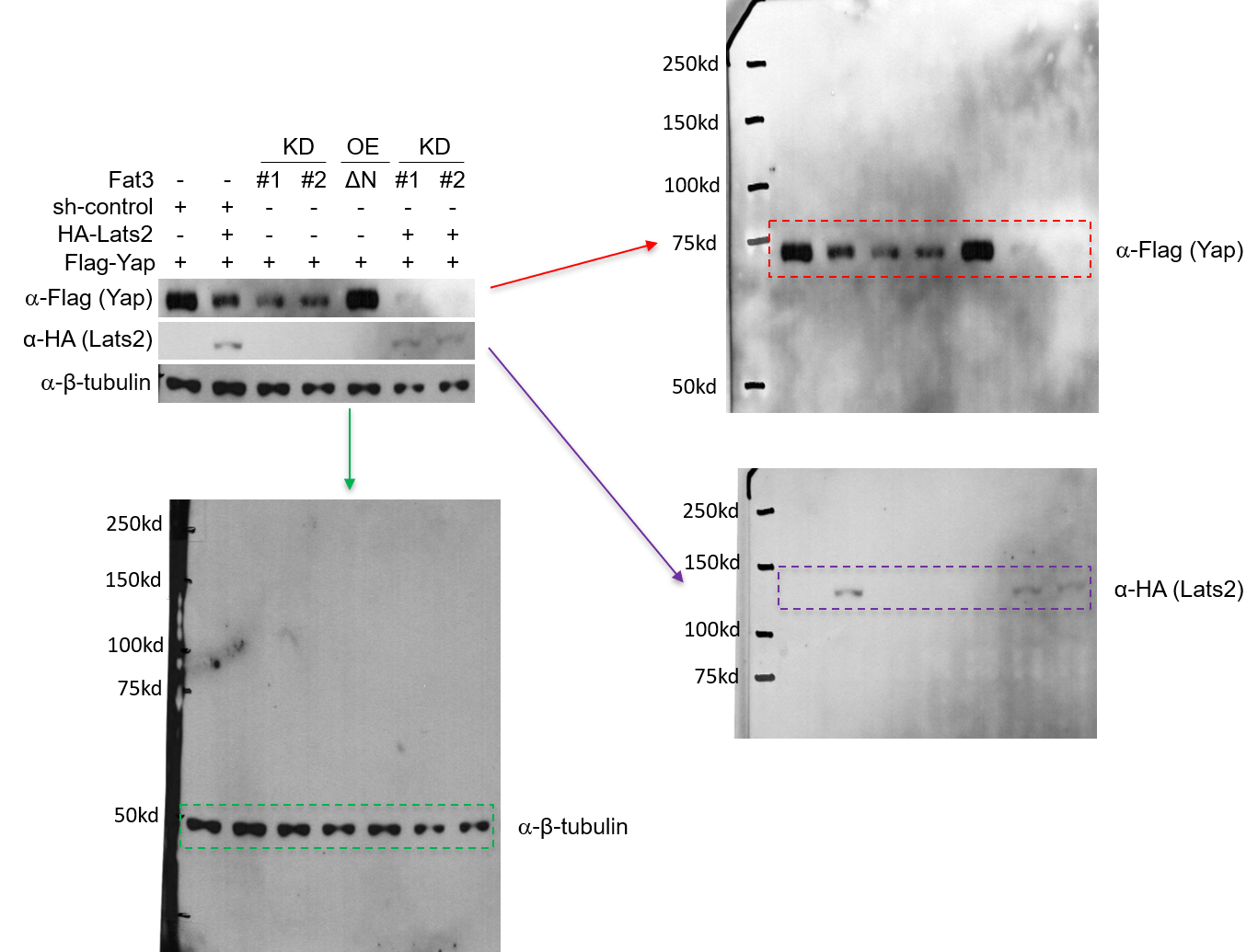


Figure S2: Full length blots of Flag-YAP, HA-Lats2 and tubulin in P19 cells (shown as cropped images in Figure 4B).


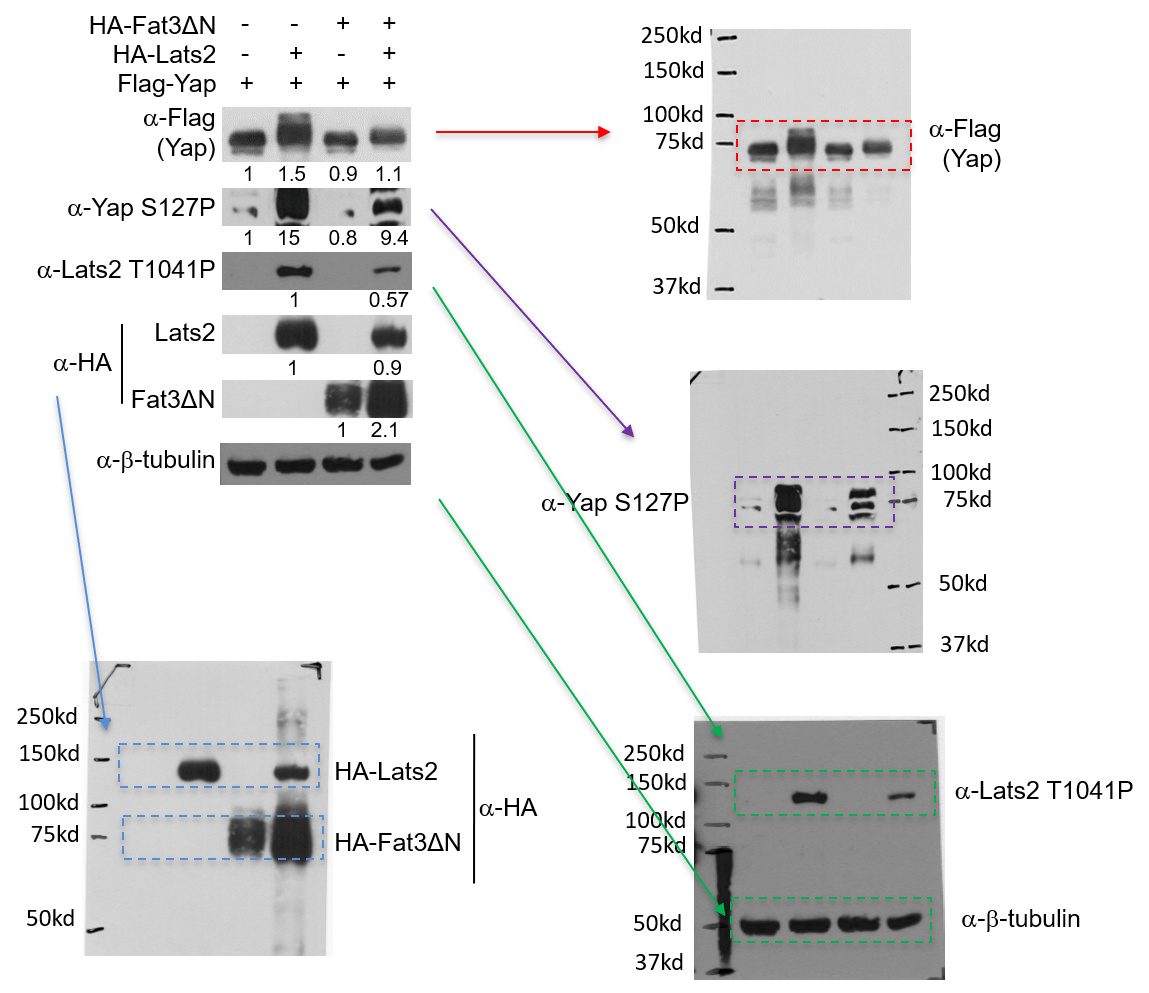


Figure S3: Full length blots of Flag-YAP, YAP S127P, Lats T1041P, HA-Lats2, HA-Fat3ΔN and tubulin in P19 cells (shown as cropped images in Figure 4D). The nitrocellulose membrane after gel transferring was cut into a small piece for Lats2 T1041P and tubulin. HA immunoblot detects both HA-tagged lats2 and HA-tagged Fat3 ΔN.


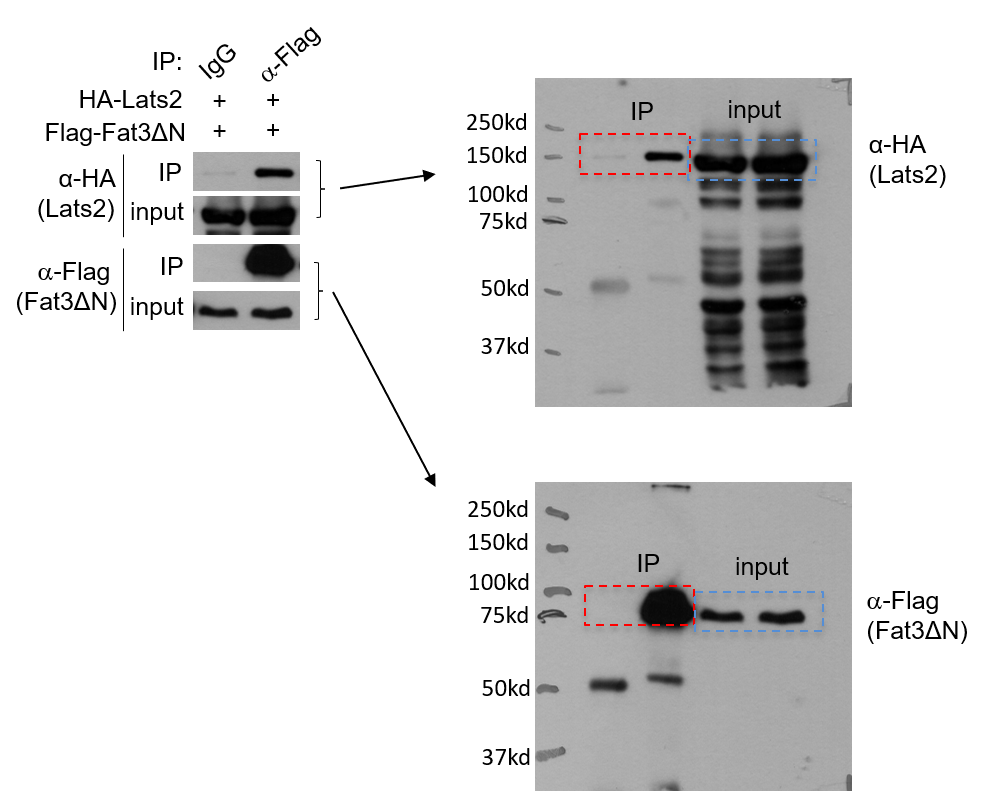


Figure S4: Full length blots of coimmunoprecipitation of HA-Lats2 and Flag-Fat3ΔN in HEK293T cells (shown as cropped images in Figure 4F).


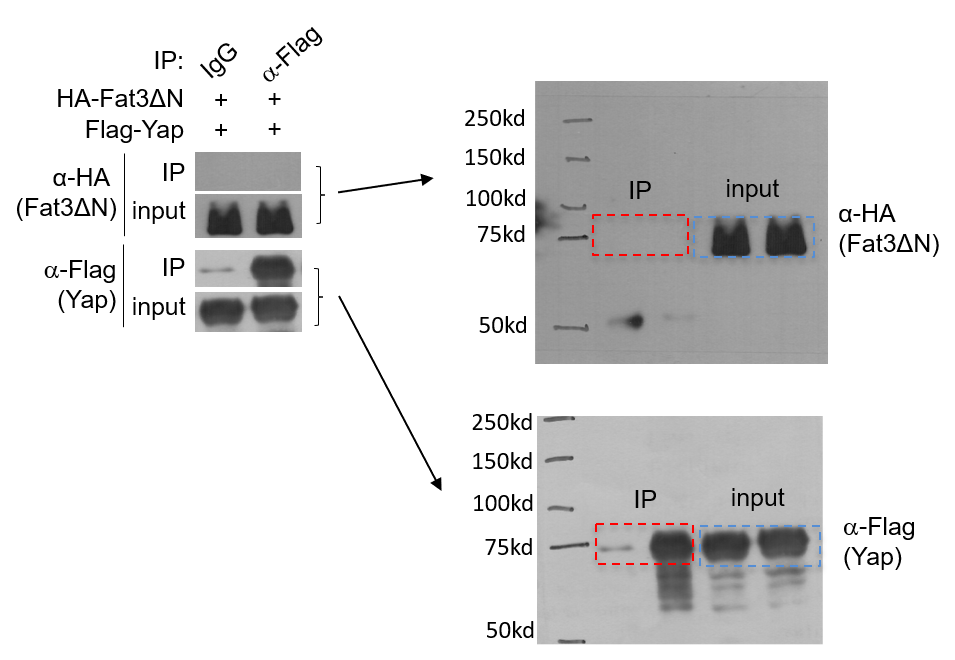


Figure S5: Full length blots of coimmunoprecipitation of Flag-YAP and HA-Fat3ΔN in HEK293T cells (shown as cropped images in Figure 4G).


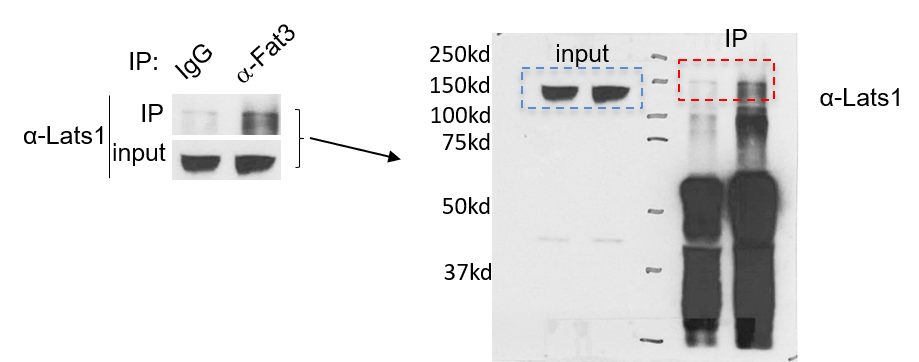


Figure S6: Full length blots of coimmunoprecipitation of Fat3 and Lats1 in mouse spinal cord extract (shown as cropped images in Figure 4H).
